# Postauricular Muscle Reflex as a Potential Objective Measure of Auditory Function in Normal-Hearing Adults

**DOI:** 10.3390/s26082524

**Published:** 2026-04-19

**Authors:** Jan-Erik Müller, Jose Luis Vargas Luna, Daniela Korth, Daniel Richter, Gerd Fabian Volk, Izet Baljić, Orlando Guntinas-Lichius

**Affiliations:** 1 Department of Otorhinolaryngology, Jena University Hospital, 07747 Jena, Germany; jan-erik.mueller@fresenius-fhs.de (J.-E.M.); daniela.korth@med.uni-jena.de (D.K.); daniel.richter2@med.uni-jena.de (D.R.); fabian.volk@med.uni-jena.de (G.F.V.); 2 MED-EL Medical Electronics, 6020 Innsbruck, Austria; jose.vargas@medel.com; 3 Department of Otorhinolaryngology, Helios Klinikum Erfurt, 99089 Erfurt, Germany; izet.baljic@helios-gesundheit.de

**Keywords:** acoustic, hearing, reflex, ear muscles, evoked potentials, objective measure

## Abstract

This study aims to establish a protocol for measuring the postauricular muscle reflex (PAMR) and to characterize both short- and mid-latency responses under controlled conditions in adults with normal hearing. PAMR electromyography was recorded in 43 adults with normal hearing. Auditory stimuli (50 ms, 80–100 dB (A)) were presented at four frequencies (500, 1000, 2000, and 4000 Hz), with systematic variation in stimulation side (ipsilateral/contralateral) and eye position (forward/rotated). The influence of these factors on PAMR amplitude and latency was analyzed using linear mixed-effects models. A short-latency PAMR (10–25 ms) was observed in all but one participant in at least one frequency. Reflex amplitude was significantly affected by stimulation side, eye position, frequency, and intensity. Contralateral stimulation produced stronger responses than ipsilateral stimulation. Additionally, a mid-latency PAMR (37–50 ms) was identified in 91% of participants, exhibiting lower amplitude and a higher detection level compared to the short-latency response. The mid-latency reflex was also significantly influenced by experimental conditions. The data shows that PAMR can be reliably recorded under controlled conditions in normal-hearing adults and that both short- and mid-latency components are influenced by auditory and oculomotor factors. These results provide us with normative data that can serve as a reference for future investigations in clinical populations, such as cochlear implant users and individuals with hearing loss.

## 1. Introduction

Auricular movement is a common feature in many mammalian species, where the extrinsic ear muscles actively orient the pinna toward sounds of interest [1]. In humans, the same reflex pathways are present but weaker, resulting in no visible ear movement. Nonetheless, subtle activity in the auricular muscles has been linked to auditory attention reflexes [1,2,3]. Specifically, reflexive responses have been documented in the postauricular muscle (PAM) and the superior auricular muscle (SAM) in a few studies with a small sample size, showing the potential in assessing auditory function. While the SAM reacts to auditory stimuli by briefly becoming suppressed, the PAM reacts with a transient increase in activity ipsilateral to the auditory stimuli [1].

Given the involuntary nature of the postauricular muscle reflex (PAMR) in response to acoustic stimuli, it provides valuable information on the function of the auditory system. Thus, it has been identified as a potential objective measure in individuals with hearing loss [4]. Objective measures to determine hearing loss are of particular use in cases where the individual being assessed cannot give subjective feedback [5,6]. Such cases commonly exist in children and individuals with special needs. Established standards for objective measurement are auditory brainstem response (ABR) and otoacoustic emissions (OAE) measurements. OAE cannot detect mild hearing loss and is sensitive to middle ear pathologies like middle ear effusion or negative pressure. ABR is time-consuming and requires patient stillness. PAMR measurements could help overcome these limitations.

The PAMR was first described as a low-threshold sonomotor response by Kiang et al. [7,8]. The postauricular reflex was interpreted to be cochleo-myogenic in origin [9,10]. The efferent component of the reflex is mediated by the facial nerve. In this sense, the PAMR is comparable to the stapedius reflex, another established objective method used in cases of hearing disorders or facial palsy. The PAMR is easily obtained using surface electrodes over the muscle behind the ear [9]. Recordings are most successful with the placement of the stimulating active electrode above the PAM and the reference electrode on the dorsal surface of the pinna [9]. The PAMR is facilitated when the muscle tone is greater, i.e., when the muscle is contracted. This PAM contraction can be achieved through the oculo-auricular phenomenon, by moving the eyes towards the ear [4,10]; likewise, general tension of the neck muscles can potentiate the response [10,11].

As early as 1969, Yoshiwe and Okudaira [12] regarded the PAMR as promising in the estimation of audiometric thresholds. Audiometric thresholds have been used clinically since the 1920s to categorize the degree and type of hearing loss. The hearing threshold is the softest subjective sound a person can hear at least 50% of the time [13]. The PAMR threshold can be near to the subjective audiometric threshold in most people with normal hearing and those with hearing loss [8,14,15,16], but it has not been employed as a routine diagnostic measure because of its perceived variability within and between test participants [4,17,18,19].

The evaluation of adequate stimulus responses of an objective measurement plays a fundamental role in its prospective use in everyday clinical practice. Thus, the present study aims to characterize the PAMR in response to a tone burst in individuals with ‘normal’ hearing, to establish a measurement protocol for the recording of the PAMR as an alternative objective measure in other populations, e.g., CI users. Furthermore, we wanted to investigate the influence of intensity, frequency, and effects of lateral eye movement on the potentiation of the reflex, and differences in the side of stimulation on the PAMR.

## 2. Methods

### 2.1. Participants and Audiometry

This study is a prospective observational study conducted at a single centre. Data were collected from March to September 2024, at the Department of Otorhinolaryngology, Jena University Hospital, Germany. The study was conducted in accordance with the Declaration of Helsinki and approved by the Ethics Committee of the University of Jena (Reg. No.: 2024-3203-BO). All participants provided written informed consent prior to inclusion in the study. Eligible participants were adults aged 18 years or older with normal hearing, defined as a pure-tone audiometry (Merz Audio, software evidENT3, Version 1.2.2.2, Merz Medizintechnik, Reutlingen, Germany, audiometric measurements following DIN EN 60645-1-2018-08), average of 15 dB HL or less in both ears at the frequencies of 500, 1000, 2000, and 4000 Hz. Additional requirements for inclusion were the absence of any diagnosed disease affecting the outer, middle, or inner ear, and no history of neurological disorders. Individuals who did not meet all these criteria were excluded from participation. The stapedius reflex thresholds were evaluated in both sides at frequencies of 500, 1000, 2000 and 4000 Hz (eTymp, also software evidENT3, Merz Medizintechnik, Reutlingen, Germany). All measurement (audiometry, stapedius reflex measurements, PAMR measurements) were performed in a standard double-wall audiology booth with a structurally isolated inner room (120a Series Hearing Test Booth; IAC Acoustics, Mönchengladbach, Germany) in the department.

### 2.2. Electromyography Recording

Following skin preparation with abrasive gel (Nuprep^®^ Skin Prep Gel; Weaver and Co., Aurora, CO, USA), a pair of self-adhesive electrodes (Löwenstein Medical; Erfurt, Germany) were placed to monitor the PAM’s electromyography (EMG) activity. One electrode was placed over the PAM, which was identified by folding the auricle forward and following the tendon back to the muscle belly. The second electrode was placed on the rear of the pinna close to the tendon [9]. The adhesive pads were cut to size with scissors prior to application. Finally, a ground electrode was placed in the middle of the forehead as reference. Prior to stimulus delivery, electrode placement and signal quality were verified through a brief facial muscle activation task. Participants were instructed to smile and pucker their lips, which reliably activates the postauricular muscle (PAM) via co-contraction of facial muscles innervated by the facial nerve. This procedure allowed rapid visual confirmation of PAM activity in both EMG channels and served as a subjective assessment of signal quality. If no discernible muscle activity was observed or if the resting EMG amplitude exceeded 50 µV—suggesting poor skin–electrode contact or elevated impedance—the skin was prepared again, and electrodes were repositioned to ensure optimal signal acquisition. All signals were acquired using a PowerLab (PL3516, AD Instruments, Dunedin, New Zealand) at 20 kS/s and the LabChart 8 Interface (AD Instruments, Dunedin, New Zealand). The EMG channels were pre-amplified using an Octal BioAmp (AD Instruments Inc., Dunedin, New Zealand) with a bandpass filter of 1 to 2000 Hz. An additional channel was dedicated to record the stimulation signal (voltage of the in-ear headphones) to synchronize the responses.

### 2.3. Stimulation

A tone burst stimulus with a total duration of 50 ms was used, consisting of a 0.5 ms linear rise time, a 49 ms plateau, and a 0.5 ms fall time. Stimuli were presented at intensities ranging from 80 to 100 dB (A), increasing in 5 dBi(A) steps every 50 repetitions. Each stimulus was delivered monaurally—first to the right ear, then to the left—using calibrated in-ear headphones (AKG, Los Angeles, CA, USA) at a repetition rate of 3 stimuli per second. Four pure-tone frequencies were evaluated: 500, 1000, 2000, and 4000 Hz.

Stimuli were calibrated using a 2 cc coupler (RA0038, GRAS Sound & Vibration, Holte, Denmark) and a sound level metre (Model 2250, Brüel & Kjær, Darmstadt, Germany) set to A-weighted fast response (LAF). A 500 ms steady pure tone was presented to allow the reading to stabilize to approximately 98% of its final value, yielding a measurement comparable to a Leq estimate. The reported stimulus levels (80–100 dB) therefore correspond to A-weighted sound pressure levels in dB (A). We acknowledge that this calibration method differs from the standard procedures recommended for tone-burst calibration, which typically rely on peak-equivalent SPL. As such, direct comparison between the present stimulus levels and those reported in studies calibrated in dB SPL or dB HL should be made with caution. A precise conversion to clinical units (e.g., dB HL or dB SPL) is not straightforward within the current setup and would require additional calibration steps not performed here.

The stimulation levels were verified at multiple time points to ensure that the intensities remained within ±1 dB (A) of the target level throughout the protocol. Each recording condition was assessed with the participant maintaining a centre-forward gaze (EF), followed by lateral eye rotation (ER) toward the side of stimulation.

### 2.4. Data Processing

All data was subsequently processed in MATLAB^®^ version R2023a (The MathWorks, Inc., Natick, MA, USA) for feature extraction. First, the EMG signals were filtered using an 8th-order Butterworth bandpass IIR filter with cut-off frequencies of 5 and 700 Hz to remove low-frequency drift and high-frequency noise.

Following filtering, individual repetitions or groups of repetitions were visually inspected and, when needed, cross-checked against session notes to identify and exclude trials containing artefacts unrelated to PAMR activity. These artefacts included eye blinks, brief head movements or shifts in posture, and electrode displacements. Artefact-contaminated trials were characterized by abrupt, high-amplitude deflections that were inconsistent with the expected PAMR morphology in terms of duration, amplitude, and timing. Such deflections typically appeared in isolation (i.e., in a single repetition), although they were often visible across multiple channels simultaneously. Electrode detachment was identified by a sudden and persistent increase in baseline noise, while voluntary muscle activity appeared as broad, unsynchronized EMG bursts unaligned with stimulus onset. In total, all repetitions from 12 stimulus–condition combinations were removed, and additional 24 individual repetitions were excluded from their respective averages. Only artefact-free repetitions were retained for averaging.

The final mean waveform, computed from the remaining clean repetitions, was used for all subsequent analyses. The use of 50 repetitions per condition further reduced minor noise components through averaging, thereby improving the signal-to-noise ratio of the PAMR.

The first peak was calculated as the maximum value within the region of 10 to 18 ms post-stimulus onset. This region of interest was defined based on the results of O’Beirne et al. [9], extended by ±20% to account for variability due to differences in setup and to allow detection of atypical or previously unreported responses. The reflex onset (latency) was calculated as the point where the signal started to increase to the first peak (zero-crossing of the first derivative). The second peak was calculated in the same way, but as the first deflection after the first peak. Finally, the peak-to-peak voltage (V_pp_) was defined as the difference in amplitude between the first and second peak.

The response detection criteria were selected based on the empirical noise characteristics of our recordings and on the canonical PAMR morphology described by O’Beirne and Patuzzi [9]. The peak-to-peak amplitude had to exceed 10 µV, which corresponds to approximately twice the typical baseline noise level after averaging 50 repetitions, ensuring that detected responses were clearly distinguishable from background activity. In addition, the first deflection was required to be a positive peak (i.e., >0 µV), consistent with the established PAMR waveform morphology and to prevent motion-related or atypical EMG fluctuations from being misclassified as reflexes.

A mid-latency response was identified within a later time window (35–50 ms). This component exhibited a biphasic morphology comparable to the short-latency PAMR. Because no standardized criteria for mid-latency PAMR detection exist in the literature, we applied the same conservative amplitude and morphology criteria to classify these responses.

In this study, the PAMR detection level was operationally defined as the lowest stimulus intensity within the tested range (80–100 dB (A)) at which the predefined response criteria were met. If a reflex was observed at 80 dB (A) this value was recorded as the detection level, as lower intensities were not evaluated in this study. On the other hand, if no PAMR was detected under a specific condition (e.g., frequency, stimulation side, eye position), that dataset was excluded from calculations of detection level, amplitude, and latency for that condition. No interpolation or imputation was applied; only observed responses were analyzed. However, the absence of responses is represented in the prevalence data reported in the Results section.

For further analysis, each PAM signal was first re-labelled based on the side of stimulation: for example, a reflex recorded from the left ear during left-ear stimulation was classified as ipsilateral, while a reflex from the left ear during right-ear stimulation was classified as contralateral. After re-labelling, all responses were pooled according to stimulation side—grouping together ipsilateral responses and contralateral responses—regardless of whether they originated from the left or right ear.

### 2.5. Statistics

Descriptive statistics were calculated for the response’s detection levels in different conditions. Additionally, Wilcoxon signed-rank tests were performed to evaluate differences in the detection levels and amplitudes for different conditions. If no detection level was found for a specific condition, it was not included in the average detection level calculation. A linear mixed model was used to assess the effect of the side of stimulation (ipsilateral vs. contralateral), eye position, frequency, intensity, gender, and age on the peak-to-peak amplitude of the reflexes detected, considering the random variability due to differences between patients in the muscle strength (random intercept). The amplitude data were normally distributed within each individual dataset; however, inter-subject variability resulted in non-normal distributions across participants. To enable meaningful comparison between subjects, reflex amplitudes were normalized using a Z-transformation within each participant, thereby standardizing the data and improving inter-dataset comparability. To complement the amplitude and latency analyses, we performed an exploratory response-presence analysis using a generalized linear mixed-effects model (GLMM) with a binomial distribution and logit link. Trial-level PAMR detectability (0 = absent, 1 = present) served as the dependent variable, while the side of stimulation (ipsilateral vs. contralateral), eye position, and frequency were investigated as categorical effects, and the amplitude was kept linear instead of categorical. Models were fitted using the *fitglme* function from MATLAB (The MathWorks, Inc., Natick, MA, USA).

A *p*-value of <0.05 was considered statistically significant.

## 3. Results

### 3.1. Hearing Characteristics of the Participants

**Figure 1:**
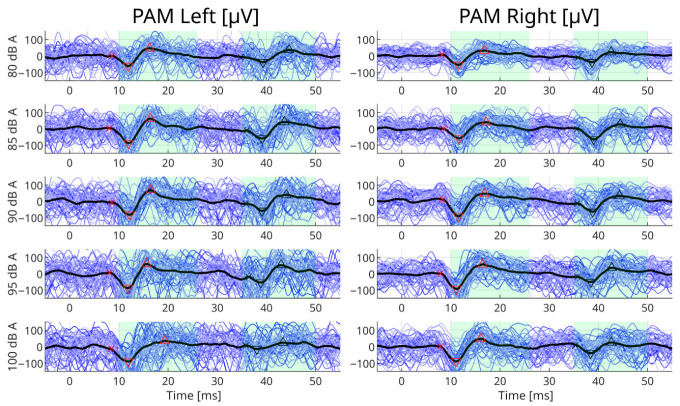
Example of response from participant P59 during right stimulation with 500 Hz and eyes rotated. The responses to stimulation with a loudness of 80, 85, 90, 95 and 100 dB (A) are shown from top to bottom. Blue lines represent the individual repetitions, and the thick black line is the average. X marks the beginning of the reflex, the triangles mark the minimum and maximum peaks of the wave, and the shadowed areas indicate the region of interest used for analyzing the signals.

**Figure 2:**
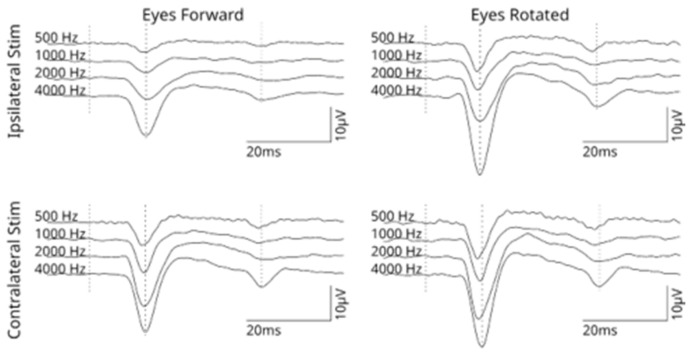
Grand average of the responses elicited in all participants using the maximum stimulation intensity ipsilateral and contralateral. Left and right columns indicate the eyes forward (EF) and eyes rotated (ER) conditions respectively. The responses to stimulation frequencies of 500, 1000, 2000 and 4000 Hz are shown from top to bottom. The dotted lines represent the stimulation onset.

In total, 43 participants were included, 17 were female and 26 were male, giving a total of 86 datasets. The median age of the participants was 33 years (range 19–63 years). Pure-tone audiometry and stapedius reflex thresholds were evaluated in both sides at frequencies of 500, 1000, 2000 and 4000 Hz, giving a total of 344 measurements. In 18 participants, the stapedius reflex was not observed in at least one frequency within the intensity range evaluated (≤100 dB HL) (Table 1). The audiometric values for all participants are shown in Supplementary Materials Figure S1, showing audiometry levels ≤20 dB HL, and the stapedius reflex threshold distribution. Figure 1 shows an example of the PAMR in one participant, and Figure 2 shows the grand average from all participants at different conditions using the maximum stimulation intensity.

### 3.2. Short-Latency Postauricular Muscle Reflex

PAMRs were assessed in both sides under the condition EF or ER, at four different frequencies and loudness levels ranging from 80 to 100 dB (A). After removing invalid repetitions, a total of 3185 stimuli combinations were applied with fifty repetitions each. Of the 3185 total stimulus combinations, 980 (30.8%) evoked an ipsilateral short-latency PAMR response, with at least one response observed in all but one participant. The same stimuli evoked contralateral responses in 1340 instances (42.1%), although these originated from a slightly smaller subset of participants (n = 39). This indicates that while contralateral responses were more frequent overall, they were distributed across fewer individuals. In total, 39 participants had both ipsilateral and contralateral short-latency PAMRs. The Supplementary Materials Figure S2 shows a more detailed prevalence of the PAMR, grouped by stimulation side, eye position, frequency, and intensity. A Wilcoxon signed-rank test was conducted to compare stimulus conditions that elicited measurable detection levels in both ears. The results indicate that contralateral detection levels (82.1 ± 4.4 dB (A)) were significantly lower than ipsilateral detection levels (84.3 ± 6.3 dB (A)), Z = 4.8, *p* < 0.001. This confirms that contralateral stimulation consistently produced lower detection levels across matched conditions.

**Figure 3:**
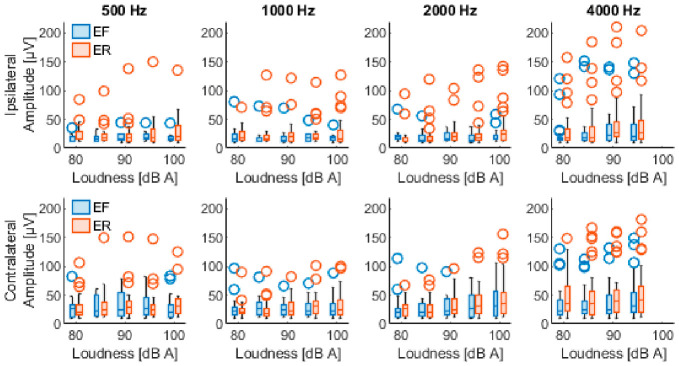
Short-latency PAMR amplitudes. (**Upper**) row shows the peak-to-peak amplitude of the responses evoked with ipsilateral stimulation at each frequency with both conditions, eyes forward (EF) and eyes rotated (ER). (**Lower**) row shows the PAM responses evoked with contralateral stimulation. Results are shown for each stimulation loudness.

**Figure 4:**
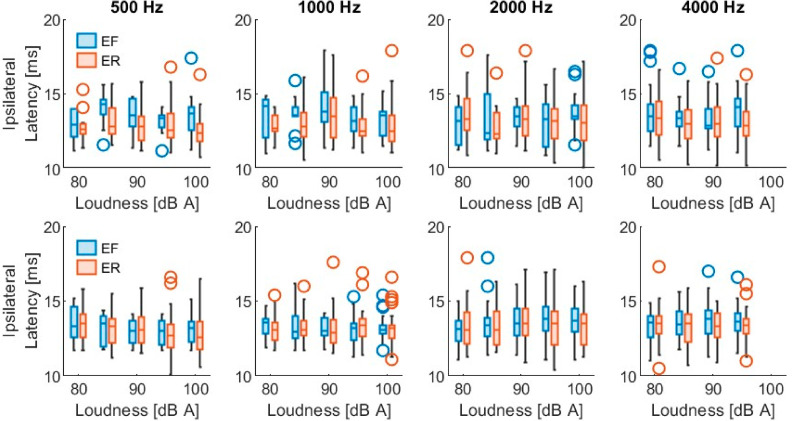
Short-latency PAMR latencies. (**Upper**) row shows the latency of the responses evoked with ipsilateral stimulation at each frequency with both conditions, eyes forward (EF) and eyes rotated (ER). (**Lower**) row shows the PAM responses evoked with contralateral stimulation. Results are shown for each stimulation loudness.

Table 2 shows the PAMR detection level (mean ± standard deviation) at each frequency, under each condition, on each side of the stimulation. Each condition was assessed on both ears of the 43 patients, giving a total of 86 datasets. The detected reflexes had peak-to-peak values from 10 µV (minimum predefined classification of a reflex) up to 271 µV, and an overall mean of 32.0 ± 28.3 µV. The latency of the first peak ranged from 10.1 up to 17.9 ms, with an overall mean of 13.3 ± 1.3 ms. Figure 3 and Supplementary Materials Table S1 show the distribution of the PAMR peak-to-peak amplitude. Similarly, Figure 4 and Supplementary Materials Table S2 show the distribution of the first peak latency.

### 3.3. Factors Influencing the Short-Latency Postauricular Muscle Reflex

Given that PAMR responses were not consistently bilateral in all participants, some subjects exhibited reflexes in only one ear, and the data from left and right were pooled; ear-level data were treated as statistically independent in this analysis. This approach reflects the localized nature of the reflex and the variability in response occurrence across ears. The results of the linear mixed model show that the eye position (*F* [1, 2308] = 80.2, *p* < 0.001), stimulation side (*F* [1, 2308] = 130.7, *p* < 0.001), frequency (*F* [3, 2308] = 103.8, *p* < 0.001), and intensity (*F* [4, 2308] = 33.4, *p* < 0.001) all had a highly significant effect on the normalized response amplitude, while gender had only a moderately significant effect (*F* [1, 2308] = 4.1, *p* = 0.044) and age had no significant effect (*F* [1, 2308] = 1.74, *p =* 0.188). More specifically, compared with 500 Hz, the 2000 Hz response (*t* [2308] = 2.7, *p* = 0.008) and 4000 Hz response (*t* [2308] = 13.7, *p* < 0.001) stimulation produced a significantly greater response. Compared with 80 dB (A), the stimuli intensity of 90, 95, and 100 dB (A) produced a significantly greater response (*p* < 0.001). The GLMM revealed significant effects of condition, frequency, intensity, and stimulation side on PAMR detectability for the short- and mid-latency response. Eyes rotated (ER) trials showed more than double the odds of eliciting a reflex compared with eyes forward (EF; β = 0.80 ± 0.08, *p* < 0.001, OR ≈ 2.22). Higher frequencies markedly increased response presence relative to 500 Hz, with odds ratios of approximately 1.27 (1000 Hz), 1.81 (2000 Hz), and 5.40 (4000 Hz). Within the tested stimulation range, each 1 dB (A) increase in intensity raised the odds of detecting a PAMR by ~3–4% (β = 0.034 ± 0.006, *p* < 0.001). Ipsilateral stimulation significantly reduced detectability compared with contralateral stimulation (β = −0.74 ± 0.08, *p* < 0.001, OR ≈ 0.48). The random intercept for subject (σ = 1.66) indicates substantial between-subject differences in baseline response probability.

### 3.4. Mid-Latency Postauricular Muscle Reflex

A short-latency PAMR was observed between 10 and 25 ms post stimuli onset. However, a second mid-latency wave was also observed in some instances between 35 and 50 ms (Figure 1). This mid-latency reflex has a similar biphasic morphology (one positive and one negative peak) to the short latency PAMR. In general, the magnitude of the mid-latency reflex was similar or smaller than the magnitude of the short-latency PAMR.

**Figure 5:**
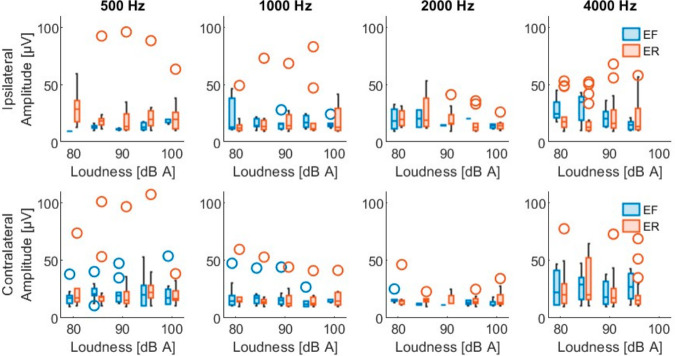
Mid-latency PAMR amplitudes. (**Upper**) row shows the peak-to-peak amplitude of the responses evoked with ipsilateral stimulation at each frequency with both conditions, eyes forward (EF) and eyes rotated (ER). (**Lower**) row shows the PAM responses evoked with contralateral stimulation. Results are shown for each stimulation loudness.

**Figure 6:**
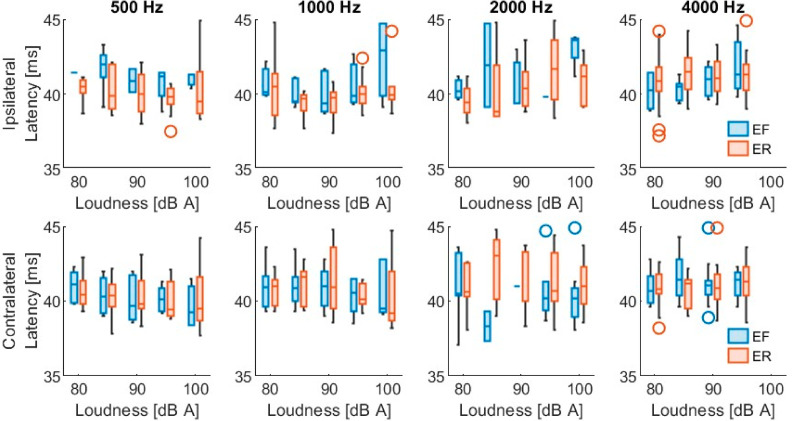
Mid-latency PAMR latencies. (**Upper**) row shows the latency of the responses evoked with ipsilateral stimulation at each frequency with both conditions, eyes forward (EF) and eyes rotated (ER). (**Lower**) row shows the PAM responses evoked with contralateral stimulation. Results are shown for each stimulation loudness.

Out of the 3185 total stimulus combinations, a mid-latency PAMR was evoked ipsilaterally in 297 instances (9.3%), originating from 29 participants. Similarly, 488 contralateral responses (15.3%) were observed, corresponding to 35 participants. In total, 23 participants had both ipsilateral and contralateral mid-latency PAMRs, while the remaining participants did not exhibit a detectable mid-latency response under the tested conditions. Like the short-latency response, a Wilcoxon signed-rank test was conducted to compare stimulus conditions that elicited measurable detection levels in both ears. The results indicate that contralateral detection levels (83.5 ± 5.6 dB (A)) were significantly lower than ipsilateral detection levels (85.9 ± 6.2 dB (A)), Z = 3.1, *p* = 0.002. Table 3 summarizes the mean detection levels for this second PAMR component. The mid-latency PAMR showed peak-to-peak values from 10 µV (minimum predefined classification of a reflex) up to 107 µV, and an overall mean of 20.8 ± 13.9 µV. The latency of its first peak ranged from 37.1 up to 44.9 ms, with an overall mean of 40.8 ± 1.5 ms. Figure 5 and Supplementary Materials Table S3 show the amplitude distribution of the mid-latency PAMR. Similarly, Figure 6 and Supplementary Materials Table S4 show the distribution of its first peak latency.

Moreover, a Wilcoxon signed-rank test comparing the detection levels of the short-latency (81.5 ± 3.7 dB (A)) and mid-latency response (85.3 ± 6.4 dB (A)) shows that over all combinations, when both responses were elicited, the short-latency responses had a significantly lower detection level compared to the mid-latency responses, Z = −9.8, *p* < 0.001. The amplitude of the responses was also compared for the ipsilateral short-latency responses (44.4 ± 40 dB (A)) and mid-latency responses (21.4 ± 15 dB (A)), showing a significant difference between both, Z = 13, *p* < 0.001. The contralateral responses also show a significant difference (Z = 18, *p* < 0.001) between the short-latency (53.0 ± 35 dB (A)) and mid-latency responses (21.9 ± 14 dB (A)).

### 3.5. Factors Influencing the Mid-Latency Postauricular Muscle Reflex

The results of the linear mixed model show that, similar to the short-latency PAMR, the mid-latency responses were strongly potentiated by the eye position (*F* [1, 767] = 11.1, *p* < 0.001), frequency (*F* [3, 767] = 56.3, *p* < 0.001), stimulation side (*F* [1, 767] = 54.8, *p* < 0.001) and intensity (*F* [4, 767] = 8.1, *p* < 0.001). On the other hand, the effect of age (*F* [1, 767] = 5.2, *p* = 0.022) and gender (*F* [1, 767] = 4.1, *p = 0.044*) were only moderately significant. More specifically, compared with 500 Hz, the 4000 Hz stimulation produced a significantly greater response (*t* [767] = 11.3, *p* < 0.001). Compared with 80 dB (A), the stimuli loudness of 95- (t [767] = 3.8, *p* = 0.001) and 100 dB (A) (t [767] = 4.7, *p* < 0.001) produced a significantly greater response. The GLMM used to examine the factors influencing detectability of the mid-latency PAMR component shows that the eye position had a strong facilitatory effect: responses were significantly more likely to be detected during eyes-rotated trials (EG), which increased the odds of a mid-latency reflex by more than twofold compared with eyes forward (β = 0.78 ± 0.10, *p* < 0.001). Frequency exerted a distinct, non-monotonic influence. Relative to 500 Hz, the likelihood of eliciting a mid-latency response was lower at 1000 Hz (β = −0.48, *p* < 0.001) and 2000 Hz (β = −0.95, *p* < 0.001), but significantly higher at 4000 Hz (β = 0.42, *p* < 0.001). In contrast to the short-latency PAMR, stimulus intensity showed no significant effect on mid-latency detectability within the 80–100 dB (A) range (β = 0.005, *p* = 0.52). Ipsilateral stimulation decreased the odds of detecting a mid-latency reflex by approximately 50% relative to contralateral stimulation (β = −0.73 ± 0.10, *p* < 0.001). The random intercept (σ = 1.45) indicates substantial inter-individual variability in baseline response probability.

## 4. Discussion

The present study establishes our measurement protocol to assess the PAMR in participants with normal hearing. Stimulation evoked a short-latency PAMR between 10 and 25 ms. A mid-latency response, with a lower amplitude and higher detection level than the short-latency PAMR, was detected in most participants. Both the early and mid-latency response amplitude was potentiated by the eye position, frequency, stimulation side, and intensity of stimulation. Higher PAMR amplitudes were observed under specific conditions, including lateral eye rotation (ER), higher stimulus frequencies, contralateral stimulation, and increased stimulus intensity.

The latency of the first peak observed in our study (mean 13.3 ± 1.3 ms) is consistent with the range reported by O’Beirne et al. [9], who described PAMR latencies between 12 and 15 ms under similar stimulation conditions. The amplitudes ranged from 10 µV up to 271 µV, with 90% of the reflexes being below 65 µV. The detection levels were relatively high compared to the thresholds found in other reports [9,20]. In addition to differences in units (dB SL or HL in earlier works vs. dB (A) in the present study), we speculate that this discrepancy may be partly explained by the limited stimulation range applied here (80–100 dB (A)). This restricted range creates a floor effect that prevents accurate estimation of true thresholds. In many cases, PAMR responses were already elicited at the lowest tested level of 80 dB (A), implying that actual thresholds may have been lower than those measured in our protocol.

Contralateral stimulation had a greater effect size than the ipsilateral stimulation and a greater number of short-latency reflexes. This was also observed in the numerical data presented, with an overall decrease in detection levels with contralateral stimulation. For mid-latency reflexes, contralateral stimulation also has a significantly greater effect size than ipsilateral stimulation. The number of reflexes increased when contralateral stimulation was applied. This supports the involvement of a reflex pathway within multiple nucleus within the brainstem [21]. While our data cannot confirm the level of engagement of the nucleus in the brainstem, some alternatives are discussed. For example, as the PAMR is considered a vestigial form of the pinna-orienting reflex, which in mammals serves to orient the auricle toward a sound source, the fact that contralateral stimulation produces stronger contractions may indicate that a greater adjustment of auricle position is required to optimize the acoustic input to the contralateral ear, thereby enhancing spatial awareness toward the sound source. Another possibility, as Doubell et al. suggest, is that the contralateral PAM contraction is closer to a vestigial startle reflex than to an attentional pinna-orienting reflex [11,22]. This would mean that the startle reflex is triggered in both ears and the function for attentional pina orientation modulates each side differently.

The observation that contralateral reflexes are stronger may facilitate PAMR detection in cochlear implant (CI) users, where electromagnetic noise from electrical stimulation and device communication could interfere with EMG recordings. Currently, the stapedius reflex is recognized as a reliable indicator of the Maximum Comfortable Level (MCL). Therefore, investigating the relationship between the PAMR, stapedius reflex, and MCL is an important direction for future research. If the PAMR can be successfully adapted for CI users, it could simplify the determination of MCL by providing an objective measure, ultimately improving CI fitting and user experience. The eye position also had a significant effect on the PAMR in the present study, like the findings of Patuzzi et al. [10], whereby looking towards the side of stimulation potentiated the reflexes. It is important to note that although the instruction given to the participants was to look towards the side of stimulation, the actual intention is that with strong eye rotation, the oculo-auricular phenomenon is triggered and the PAM becomes contracted. In this sense, turning the head completely or a slight gaze towards the stimulation side has no effect on the response. Therefore, pre-contracting the PAM has a significant impact on the results.

Because amplitude and latency analyses necessarily include only trials with a measurable waveform, the present study may overrepresent conditions in which PAMR responses were more readily elicited. To address this limitation, we conducted an exploratory logistic mixed-effects analysis modelling PAMR detectability directly. This analysis confirmed the strong influence of eye position, stimulation side, frequency, and intensity on the likelihood of eliciting a reflex, consistent with the prevalence patterns reported earlier. Although detectability modelling was not a primary aim of the current work, these results illustrate the value of analyzing response presence alongside waveform morphology. Future studies—particularly those involving populations with reduced or inconsistent PAMR responses—should incorporate full logistic-regression frameworks to characterize behaviour and clinical utility more comprehensively.

The detection of a mid-latency response evoked in 90% of the participants was unexpected—to our knowledge it has not been reported previously. In O’Beirne’s [9] and Yoshie and Okudaira’s work [22], some figures at similar intensities to ours show a resemblance to this mid-latency wave; however, they were not explicitly described. Despite our setup not being specifically designed to study this component, several observations support its physiological origin rather than artefact: it exhibited a stable onset latency, a higher detection level (characteristic of secondary responses), and a clear biphasic morphology. Moreover, the logistic mixed-effects analysis demonstrates that mid-latency detectability is strongly influenced by the same experimental factors as the short-latency PAMR—eye rotation and contralateral stimulation substantially increased response presence—while showing a distinct, non-linear pattern across frequencies and no significant sensitivity to intensity within the tested range. These characteristics suggest a related but functionally distinct reflex pathway, possibly involving a longer polysynaptic circuit. The underlying neural mechanisms of this component remain uncertain as current data cannot support any conclusions. Therefore, further dedicated studies will be required to determine the precise neural mechanisms underlying this mid-latency response and its relationship to the established PAMR pathway. A possible research line could be to study shared pathways with other responses known to be acoustically evoked and to have similar latency ranges, such as the startle-related pathways that produce myogenic activity within similar latency ranges (45–75 ms) and overlap with components of the blink reflex [21,23]. A contribution from auditory mid-latency responses (AMLR) cannot be fully excluded; however, the markedly different morphology and higher amplitudes observed here argue against a direct overlap.

This study has some limitations that should be considered when interpreting the results. First, since the primary goal was to assess the PAMR as a potential marker for comfortable hearing levels, the stimulation range used (80 to 100 dB (A)) was relatively narrow and did not include enough sub-threshold or near-threshold levels. Consequently, detection levels may be higher than actual thresholds for some participants, as responses could occur at lower intensities. Therefore, follow-up studies should include a wider stimulation range.

A second limitation concerns the calibration of the acoustic stimuli. In this study, stimulus levels were referenced to dB (A) using an A-weighted fast-response (LAF) measurement rather than the recommended peak-equivalent SPL. Although the use of a 500 ms steady tone allowed the measurements to approximate an Leq-type estimate, this approach is not directly comparable to standard clinical calibrations (e.g., dB HL or dB SPL). Consequently, the absolute stimulation levels reported here cannot be assumed to map directly onto clinical audiometric scales or to correspond precisely with thresholds reported in previous PAMR studies. However, because all conditions and stimulus manipulations were calibrated using the same procedure, the relative comparisons within the study—such as the effects of stimulation side, frequency, intensity, and eye position on detection levels, amplitude, latency, and response presence—remain fully valid. These predictors were evaluated under internally consistent acoustic conditions, and their estimated effects are therefore not affected by the calibration scheme. Future studies should nevertheless adopt ISO-compliant peak-equivalent SPL calibration or provide explicit conversions to clinical units to facilitate direct comparison with established literature and audiological practice.

Third, although gender was statistically significant for both short- and mid-latency PAMR responses—and age for the mid-latency response—the effect sizes were modest compared with those of the main experimental factors. These findings should be interpreted with caution. In our mixed-effects framework, predictors such as eye position, frequency, stimulation side, and intensity benefit from a large number of repeated within-subject observations and therefore have strong statistical power. In contrast, age and gender vary only between participants and are effectively evaluated at the level of the 43 individuals included in the study, resulting in lower power to detect small demographic effects. This distinction explains why demographic predictors show weaker and less consistent significance despite robust effects of the experimental manipulations. Future studies with larger and more balanced participant samples may help clarify the true relevance and stability of age- and gender-related influences on the PAMR.

The pros and cons of the PAMR in comparison to other established objective neurotological measurement tools are summarized in Table 4. Like for the vestibular evoked myogenic potentials (VEMPs), the EMG signal can also be used for feedback of the muscle activity directly to the patient (VEMP: usually from sternocleidomastoid muscle; PAMR: eye position). The patient needs (like for the VEMP) measurements, clear instructions and ongoing verbal encouragement, helping to maintain steady contraction. The risk of EMG cross-over is limited due to the relatively isolated position of the muscle and only myogenic artefacts originating from general movements such as blinking or chewing could contaminate individual repetitions. Furthermore, multiple recordings are taken. Trials with a too low (or inconsistent) EMG activity should be rejected. Furthermore, as with the stapedius reflex, patients must be informed about the loudness of the measurement (threshold for both methods: 70–100 dB HL).

Finally, it is important to acknowledge that the PAMR is not present in 100% of individuals. Previous anatomical studies have reported complete absence of the postauricular muscle in approximately 5% of cadaver specimens [24], while early electrophysiological studies observed absent reflexes in up to 20% of ears tested, with 7% of participants showing no response in either ear [25]. In our study, short-latency responses were absent in one subject (~2%), and mid-latency responses were absent in approximately 19% of the cohort. These false negatives limit the maximal achievable sensitivity of the PAMR as an objective measure of auditory function in a clinical context. Furthermore, because our stimulation range (80–100 dB (A)) imposed a floor effect, threshold estimation was constrained, and the present data cannot be used to evaluate PAMR sensitivity to mild-to-moderate hearing loss. Future studies incorporating broader intensity ranges and hearing-impaired populations will be necessary to determine the true sensitivity and specificity of the PAMR.

Despite this limited prevalence, the superficial location of the muscle and the large amplitude of the reflex make the PAMR a promising candidate for integration into future cochlear implant systems with closed-loop calibration, where accessibility and signal robustness are critical.

## 5. Conclusions

The PAMR can be reliably recorded under controlled conditions in normal-hearing participants, and the amplitude and latency of the short-latency response are comparable to previous reports. We also characterize a mid-latency PAMR component, which represents a novel finding. Moving forward, the use of the PAMR as an objective marker in auditory function—particularly in cochlear implant users—will require careful evaluation of how factors such as eye position, frequency, stimulation side, and intensity influence reflex detection. These findings provide normative data and practical guidance for future research aimed at integrating the PAMR into auditory rehabilitation strategies.

## Figures and Tables

**Table 1:** Audiometry and stapedius reflex measurements.

Frequency (Hz)	Pure Tone Audiometry (dB HL) Mean ± SD	Stapedius Reflex (dB HL) Threshold Mean ± SD
500	7.5 ± 4.8 (n = 86)	90.2 ± 6.4 (n = 80)
1000	8.0 ± 4.9 (n = 86)	91.5 ± 7.0 (n = 78)
2000	8.3 ± 5.5 (n = 86)	92.8 ± 6.5 (n = 68)
4000	11.7 ± 6.8 (n = 86)	92.2 ± 7.3 (n = 71)

SD = standard deviation, n = number of successful measurements.

**Table 2:** Detection level of the PAMR detected with ipsilateral and contralateral stimulation under the condition eyes forward and with the eyes rotated in the direction of the stimulus.

Frequency (Hz)	Ipsilateral Detection Level (dB (A))		Contralateral Detection Level (dB (A))	
	Eyes Forward Mean ± SD	Eyes Rotated Mean ± SD	Eyes Forward Mean ± SD	Eyes Rotated Mean ± SD
500	85 ± 6.6 (n = 18)	85.4 ± 6.4 (n = 39)	84.1 ± 6.7 (n = 28)	82.7 ± 5.1 (n = 39)
1000	83.5 ± 5.4 (n = 20)	85.4 ± 6.6 (n = 39)	83 ± 6.3 (n = 38)	83.2 ± 6.3 (n = 47)
2000	85.5 ± 7.3 (n = 31)	87.2 ± 7.1 (n = 49)	84.3 ± 6.7 (n = 53)	84 ± 6.2 (n = 50)
4000	82.1 ± 4 (n = 49)	83.1 ± 5 (n = 70)	81 ± 2.6 (n = 52)	82.1 ± 4 (n = 57)

SD = standard deviation, n = number of PAMRs that achieved a reflex from a maximum of 86 (2 per participant).

**Table 3:** Detection level of the mid-latency PAMR detected with ipsilateral and contralateral stimulation under the condition eyes forward and with the eyes rotated in the direction of the stimulus.

Frequency (Hz)	Ipsilateral Detection Level (dB (A))		Contralateral Detection Level (dB (A))	
	Eyes Forward Mean ± SD	Eyes Rotated Mean ± SD	Eyes Forward Mean ± SD	Eyes Rotated Mean ± SD
500	84.4 ± 1.7 (n = 9)	87.5 ± 6.5 (n = 22)	81.3 ± 3 (n = 15)	84.8 ± 6.6 (n = 28)
1000	83.1 ± 2.6 (n = 8)	86.2 ± 7.4 (n = 20)	81.8 ± 3 (n = 17)	86.1 ± 6.7 (n = 23)
2000	91.5 ± 9.1 (n = 10)	89.7 ± 6.6 (n = 19)	85.7 ± 7.8 (n = 15)	88.2 ± 8.5 (n = 20)
4000	87.5 ± 7.2 (n = 10)	84.4 ± 4.6 (n = 33)	83.5 ± 4.8 (n = 30)	83.5 ± 5.3 (n = 37)

SD = standard deviation, n = number of PAMs that achieved a reflex from a maximum of 86 (2 per participant).

**Table 4:** Comparison of the pros and cons of the PAMR compared to other objective neurotological tests.

Test	Pros	Cons
PAMR (Postauricular Muscle Reflex)	Relatively large and isolated muscle (compared to stapedius muscle) Easily accessible with non-invasive techniques and implantable methods (near CI final position) High contralateral response allows for contralateral CI activation with minimal electrical contamination	Requires patient cooperation (muscle activation) Muscle might be cut during CI surgeries Further research necessary to associate it with key acoustic metrics like hearing level or most comfortable levels (CI fitting)
OAE (Otoacoustic Emissions)	Quick and easy Non-invasive, no electrodes Ideal for newborn screening Sensitive to outer hair cell function	Not a true hearing threshold measure Miss neural disorders (e.g., Auditory Neuropathy Spectrum Disorder) Affected by middle ear issues Poor for mild hearing loss
ABR (Auditory Brainstem Response)	Objective estimate of hearing thresholds Assesses auditory nerve & brainstem pathways Useful for infants/uncooperative patients Can detect retrocochlear pathology	Time-consuming May require sedation Limited frequency specificity Does not assess cortical processing or perception
SR (Stapedius Reflex)	Quick, objective test Assesses reflex arc (middle ear → brainstem) Helps identify site of lesion (conductive, cochlear, retrocochlear) No sedation required	Not a direct measure of hearing thresholds Can be absent in normal hearing (variability) Affected by middle ear pathology Limited diagnostic specificity alone
VEMP (Vestibular Evoked Myogenic Potentials)	Assesses vestibular (otolith) function Helps diagnose vestibular disorders Non-invasive Provides side-specific info	Not a hearing test (vestibular only) Requires patient cooperation (muscle activation) Sensitive to electrode placement Limited clinical availability in some settings

## Data Availability

The datasets used and analyzed during the current study are available from the corresponding author on reasonable request. Correspondence and requests for materials should be addressed to O.G.L.

## Supplementary Materials

The following supporting information can be downloaded at: https://www.mdpi.com/article/10.3390/s26082524/s1, Table S1: Short-Latency PAMR peak-to-peak voltage, the difference in amplitude between the first and second peak, with ipsilateral and contralateral stimulation under the condition eyes forward and with the eyes rotated; Table S2: Short-latency PAMR latency to first peak with ipsilateral and contralateral stimulation under the condition eyes forward and with the eyes rotated; Table S3: Mid-latency PAMR peak-to-peak voltage, the difference in amplitude between the first and second peak, with ipsilateral and contralateral stimulation under the condition eyes forward and with the eyes rotated; Table S4: Mid-latency PAMR latency to first peak (ms) with ipsilateral and contralateral stimulation under the condition eyes forward and with the eyes rotated; Figure S1: Hearing condition of the subjects. (A,B) show the results of the pure tone audiometry. (C,D) show the results of the stapedius reflex threshold; Figure S2. Prevalence of the PAMR across the most significant variables.
