# Supplementary material for: Postauricular Muscle Reflex as a Potential Objective Measure of Auditory Function in Normal-Hearing Adults
Source: Sensors (Basel). 2026 Apr 19;26(8):2524. doi: 10.3390/s26082524 (PMC13120091; doi:10.3390/s26082524)

Supplementary Material

Postauricular Muscle Reflex as a Potential Objective Measure of Auditory Function in Normal Hearing Adults

Jan-Erik Müller[1], José Luis Vargas Luna[2], Daniela Korth[1], Daniel Richter[1], Gerd Fabian Volk[1], Izet Baljić[3]. Orlando Guntinas-Lichius[1]*

[1] Department of Otorhinolaryngology, Jena University Hospital, 07747 Jena, Germany; jan-erik.mueller@fresenius-fhs.de (J.-E.M.); daniela.korth@med.uni-jena.de (D.K.); daniel.richter2@med.uni-jena.de (D.R.); fabian.volk@med.uni-jena.de (G.F.V.)

[2] MED-EL Medical Electronics, 6020 Innsbruck, Austria; jose.vargas@medel.com

[3] Department of Otorhinolaryngology, Helios Klinikum Erfurt, 99089 Erfurt, Germany; izet.baljic@helios-gesundheit.de

* Correspondence: orlando.guntinas@med.uni-jena.de; Tel.: +49-3641-9329301

Supplementary Tables S1-S4

Supplementary Figure S1-S2

Supplementary Table S1. Short-Latency PAMR peak-to-peak voltage, the difference in amplitude between the first and second peak, with ipsilateral and contralateral stimulation under the condition eyes forward and with the eyes rotated.

Loudness		Ipsilateral Amplitude (µV)		Contralateral Amplitude (µV)	
		**Eyes forward** Mean ± SD	**Eyes rotated** Mean ± SD	**Eyes forward** Mean ± SD	**Eyes rotated** Mean ± SD
**500 Hz**	**80 dB(A)**	17.3 ± 8.6 (n = 8)	24.3 ± 17.7 (n = 20)	32.8 ± 25.2 (n = 18)	30.9 ± 24.1 (n = 29)
	**85 dB(A)**	17.2 ± 6.6 (n = 12)	24.0 ± 18.4 (n = 24)	28.8 ± 16.5 (n = 17)	34.4 ± 29.0 (n = 29)
	**90 dB(A)**	19.1 ± 9.6 (n = 12)	25.0 ± 24.2 (n = 29)	30.9 ± 22.1 (n = 21)	36.4 ± 29.1 (n = 32)
	**95 dB(A)**	21.1 ± 9.1 (n = 13)	25.4 ± 25.1 (n = 35)	32.3 ± 20.2 (n = 19)	33.0 ± 27.4 (n = 35)
	**100 dB(A)**	18.7 ± 8.3 (n = 13)	29.1 ± 25.6 (n = 31)	29.7 ± 25.2 (n = 25)	37.5 ± 27.8 (n = 32)
**1000 Hz**	**80 dB(A)**	22.3 ± 18.8 (n = 13)	23.2 ± 14.5 (n = 19)	25.4 ± 17.2 (n = 29)	23.9 ± 14.1 (n = 34)
	**85 dB(A)**	18.5 ± 16.2 (n = 14)	25.7 ± 26.9 (n = 23)	28.5 ± 15.5 (n = 28)	24.1 ± 16.8 (n = 36)
	**90 dB(A)**	18.9 ± 14.3 (n = 16)	26.9 ± 25.1 (n = 23)	27.1 ± 12.6 (n = 28)	28.5 ± 16.5 (n = 37)
	**95 dB(A)**	20.6 ± 10.6 (n = 11)	25.4 ± 21.4 (n = 29)	27.8 ± 15.5 (n = 30)	34.7 ± 22.0 (n = 33)
	**100 dB(A)**	18.6 ± 7.7 (n = 12)	28.7 ± 27.2 (n = 31)	29.4 ± 21.5 (n = 35)	33.6 ± 25.4 (n = 43)
**2000 Hz**	**80 dB(A)**	21.3 ± 13.0 (n = 16)	22.6 ± 21.0 (n = 18)	28.7 ± 29.0 (n = 34)	28.8 ± 16.9 (n = 31)
	**85 dB(A)**	18.5 ± 11.4 (n = 20)	23.6 ± 24.5 (n = 24)	29.5 ± 24.7 (n = 31)	27.6 ± 19.8 (n = 36)
	**90 dB(A)**	20.5 ± 8.2 (n = 13)	26.0 ± 21.8 (n = 26)	33.1 ± 22.8 (n = 33)	34.7 ± 24.5 (n = 39)
	**95 dB(A)**	18.4 ± 8.7 (n = 17)	26.4 ± 27.7 (n = 37)	32.6 ± 22.5 (n = 36)	40.3 ± 31.6 (n = 41)
	**100 dB(A)**	21.1 ± 11.1 (n = 24)	31.6 ± 28.7 (n = 45)	39.1 ± 29.6 (n = 45)	45.3 ± 35.1 (n = 49)
**4000 Hz**	**80 dB(A)**	21.6 ± 22.0 (n = 36)	30.2 ± 29.6 (n = 45)	36.4 ± 32.8 (n = 45)	49.3 ± 38.2 (n = 42)
	**85 dB(A)**	27.3 ± 32.7 (n = 31)	31.3 ± 32.9 (n = 55)	36.0 ± 27.4 (n = 44)	48.0 ± 43.4 (n = 49)
	**90 dB(A)**	33.4 ± 31.4 (n = 30)	41.2 ± 40.0 (n = 51)	36.4 ± 27.1 (n = 47)	49.2 ± 39.4 (n = 50)
	**95 dB(A)**	32.9 ± 29.7 (n = 40)	42.2 ± 45.5 (n = 64)	44.0 ± 36.9 (n = 46)	48.2 ± 38.9 (n = 52)

SD = standard deviation, n = number

Supplementary Table S2. Short-latency PAMR latency to first peak with ipsilateral and contralateral stimulation under the condition eyes forward and with the eyes rotated.

Loudness		Ipsilateral Latency (ms)		Contralateral Latency (ms)	
		**Eyes forward** Mean ± SD	**Eyes rotated** Mean ± SD	**Eyes forward** Mean ± SD	**Eyes rotated** Mean ± SD
500 Hz	80 dB(A)	12.9 ± 1.0 (n = 8)	12.9 ± 1.1 (n = 20)	13.4 ± 1.2 (n = 18)	13.4 ± 1.2 (n = 29)
	85 dB(A)	14.0 ± 1.1 (n = 12)	13.2 ± 1.2 (n = 24)	13.1 ± 1.0 (n = 17)	13.2 ± 1.1 (n = 29)
	90 dB(A)	13.6 ± 1.0 (n = 12)	12.8 ± 1.1 (n = 29)	12.9 ± 0.8 (n = 21)	13.1 ± 1.0 (n = 32)
	95 dB(A)	13.1 ± 0.8 (n = 13)	12.9 ± 1.3 (n = 35)	12.9 ± 0.8 (n = 19)	13.0 ± 1.4 (n = 35)
	100 dB(A)	13.6 ± 1.5 (n = 13)	12.5 ± 1.1 (n = 31)	13.1 ± 0.8 (n = 25)	12.8 ± 1.3 (n = 32)
1000 Hz	80 dB(A)	13.4 ± 1.4 (n = 13)	12.9 ± 0.8 (n = 19)	13.3 ± 0.8 (n = 29)	13.2 ± 0.9 (n = 34)
	85 dB(A)	13.7 ± 1.3 (n = 14)	13.1 ± 1.4 (n = 23)	13.3 ± 1.2 (n = 28)	13.2 ± 1.0 (n = 36)
	90 dB(A)	14.1 ± 1.9 (n = 16)	13.7 ± 1.8 (n = 23)	13.2 ± 0.7 (n = 28)	13.2 ± 1.2 (n = 37)
	95 dB(A)	13.3 ± 1.1 (n = 11)	12.8 ± 1.1 (n = 29)	13.1 ± 0.9 (n = 30)	13.2 ± 1.2 (n = 33)
	100 dB(A)	13.3 ± 1.1 (n = 12)	13.0 ± 1.6 (n = 31)	13.2 ± 0.9 (n = 35)	13.2 ± 1.1 (n = 43)
2000 Hz	80 dB(A)	13.1 ± 1.3 (n = 16)	13.9 ± 1.9 (n = 18)	13.1 ± 1.0 (n = 34)	13.4 ± 1.5 (n = 31)
	85 dB(A)	13.5 ± 2.1 (n = 20)	12.9 ± 1.4 (n = 24)	13.4 ± 1.4 (n = 31)	13.2 ± 1.2 (n = 36)
	90 dB(A)	13.4 ± 1.0 (n = 13)	13.5 ± 1.8 (n = 26)	13.6 ± 1.3 (n = 33)	13.5 ± 1.3 (n = 39)
	95 dB(A)	13.2 ± 1.5 (n = 17)	13.1 ± 1.5 (n = 37)	13.8 ± 1.5 (n = 36)	13.4 ± 1.5 (n = 41)
	100 dB(A)	13.8 ± 1.2 (n = 24)	13.3 ± 1.7 (n = 45)	13.7 ± 1.3 (n = 45)	13.4 ± 1.3 (n = 49)
4000 Hz	80 dB(A)	13.6 ± 1.6 (n = 36)	13.4 ± 1.4 (n = 45)	13.5 ± 0.9 (n = 45)	13.4 ± 1.2 (n = 42)
	85 dB(A)	13.4 ± 1.1 (n = 31)	12.9 ± 1.3 (n = 55)	13.5 ± 0.9 (n = 44)	13.3 ± 1.1 (n = 49)
	90 dB(A)	13.3 ± 1.2 (n = 30)	13.2 ± 1.4 (n = 51)	13.7 ± 1.2 (n = 47)	13.3 ± 1.0 (n = 50)
	95 dB(A)	13.9 ± 1.4 (n = 40)	13.2 ± 1.4 (n = 64)	13.6 ± 0.9 (n = 46)	13.4 ± 1.0 (n = 52)

SD = standard deviation, n = number

Supplementary Table S3. Mid-latency PAMR peak-to-peak voltage, the difference in amplitude between the first and second peak, with ipsilateral and contralateral stimulation under the condition eyes forward and with the eyes rotated.

Loudness		Ipsilateral Amplitude (µV)		Contralateral Amplitude (µV)	
		Eyes forward<br>Mean ± SD	Eyes rotated<br>Mean ± SD	Eyes forward<br>Mean ± SD	Eyes rotated<br>Mean ± SD
500 Hz	80 dB(A)	10.2 ± 0.0<br>(n = 1)	30.2 ± 17.9<br>(n = 5)	18.0 ± 7.4<br>(n = 12)	22.0 ± 15.0<br>(n = 16)
	85 dB(A)	13.6 ± 2.1<br>(n = 9)	25.3 ± 23.9<br>(n = 10)	22.2 ± 8.0<br>(n = 11)	23.1 ± 21.6<br>(n = 18)
	90 dB(A)	11.7 ± 0.9<br>(n = 4)	22.0 ± 21.2<br>(n = 16)	21.2 ± 10.9<br>(n = 11)	22.0 ± 19.5<br>(n = 19)
	95 dB(A)	13.9 ± 3.4<br>(n = 7)	25.6 ± 20.3<br>(n = 13)	23.5 ± 13.9<br>(n = 10)	27.7 ± 21.8<br>(n = 18)
	100 dB(A)	18.9 ± 2.4<br>(n = 3)	23.9 ± 17.3<br>(n = 9)	21.7 ± 12.7<br>(n = 10)	20.1 ± 7.2<br>(n = 17)
1000 Hz	80 dB(A)	23.7 ± 19.9<br>(n = 3)	16.9 ± 12.7<br>(n = 9)	18.0 ± 10.9<br>(n = 12)	18.9 ± 14.6<br>(n = 10)
	85 dB(A)	16.8 ± 4.5<br>(n = 7)	19.9 ± 18.2<br>(n = 11)	17.9 ± 8.1<br>(n = 14)	17.5 ± 11.9<br>(n = 11)
	90 dB(A)	16.4 ± 5.8<br>(n = 7)	22.4 ± 19.6<br>(n = 8)	17.5 ± 9.4<br>(n = 11)	16.6 ± 9.3<br>(n = 14)
	95 dB(A)	18.2 ± 6.7<br>(n = 3)	21.3 ± 22.1<br>(n = 12)	14.3 ± 6.5<br>(n = 6)	16.6 ± 9.7<br>(n = 9)
	100 dB(A)	16.5 ± 4.4<br>(n = 6)	19.9 ± 13.1<br>(n = 6)	15.0 ± 1.3<br>(n = 5)	19.1 ± 10.9<br>(n = 7)
2000 Hz	80 dB(A)	20.6 ± 11.5<br>(n = 3)	21.4 ± 8.2<br>(n = 4)	16.1 ± 3.5<br>(n = 9)	17.4 ± 11.0<br>(n = 9)
	85 dB(A)	20.7 ± 10.8<br>(n = 2)	26.3 ± 18.8<br>(n = 4)	12.0 ± 1.3<br>(n = 2)	15.6 ± 4.2<br>(n = 6)
	90 dB(A)	14.8 ± 0.4<br>(n = 3)	20.9 ± 10.3<br>(n = 8)	11.1 ± 0.0<br>(n = 2)	15.6 ± 5.3<br>(n = 7)
	95 dB(A)	21.0 ± 0.0<br>(n = 2)	15.7 ± 7.9<br>(n = 16)	13.4 ± 2.0<br>(n = 5)	15.0 ± 4.0<br>(n = 12)
	100 dB(A)	14.3 ± 2.2<br>(n = 4)	15.2 ± 4.5<br>(n = 10)	12.8 ± 2.8<br>(n = 8)	16.5 ± 7.1<br>(n = 14)
4000 Hz	80 dB(A)	28.1 ± 11.9<br>(n = 4)	23.2 ± 15.3<br>(n = 14)	25.8 ± 14.2<br>(n = 18)	24.8 ± 16.9<br>(n = 24)
	85 dB(A)	30.2 ± 13.5<br>(n = 5)	18.9 ± 13.6<br>(n = 21)	27.9 ± 13.1<br>(n = 18)	30.2 ± 18.6<br>(n = 21)
	90 dB(A)	22.0 ± 9.5<br>(n = 6)	23.5 ± 17.6<br>(n = 16)	22.8 ± 10.9<br>(n = 20)	23.5 ± 16.3<br>(n = 29)
	95 dB(A)	15.4 ± 3.7<br>(n = 10)	22.3 ± 15.9<br>(n = 16)	27.6 ± 11.4<br>(n = 15)	19.4 ± 13.0<br>(n = 28)

SD = standard deviation, n = number

Supplementary Table S4. Mid-latency PAMR latency to first peak (ms) with ipsilateral and contralateral stimulation under the condition eyes forward and with the eyes rotated.

Loudness		Ipsilateral Latency (ms)		Contralateral Latency (ms)	
		**Eyes forward** Mean ± SD	**Eyes rotated** Mean ± SD	**Eyes forward** Mean ± SD	**Eyes rotated** Mean ± SD
**500 Hz**	**80 dB(A)**	41.4 ± 0.0 (n = 1)	40.3 ± 1.0 (n = 5)	41.0 ± 0.9 (n = 12)	40.7 ± 1.0 (n = 16)
	**85 dB(A)**	41.8 ± 1.3 (n = 9)	40.2 ± 1.3 (n = 10)	40.4 ± 1.2 (n = 11)	40.3 ± 1.3 (n = 18)
	**90 dB(A)**	40.9 ± 0.9 (n = 4)	40.2 ± 1.4 (n = 16)	40.2 ± 1.4 (n = 11)	40.3 ± 1.2 (n = 19)
	**95 dB(A)**	40.6 ± 1.0 (n = 7)	39.7 ± 0.9 (n = 13)	40.1 ± 0.8 (n = 10)	40.0 ± 1.2 (n = 18)
	**100 dB(A)**	41.0 ± 0.5 (n = 3)	40.2 ± 2.1 (n = 9)	39.6 ± 1.3 (n = 10)	40.2 ± 1.8 (n = 17)
**1000 Hz**	**80 dB(A)**	40.7 ± 1.3 (n = 3)	40.5 ± 2.2 (n = 9)	41.0 ± 1.5 (n = 12)	40.8 ± 1.1 (n = 10)
	**85 dB(A)**	40.1 ± 0.9 (n = 7)	39.4 ± 0.8 (n = 11)	41.0 ± 1.3 (n = 14)	41.0 ± 1.4 (n = 11)
	**90 dB(A)**	40.0 ± 1.4 (n = 7)	39.4 ± 1.1 (n = 8)	40.8 ± 1.4 (n = 11)	41.5 ± 2.1 (n = 14)
	**95 dB(A)**	40.6 ± 1.8 (n = 3)	40.1 ± 1.1 (n = 12)	40.3 ± 1.2 (n = 6)	40.4 ± 0.8 (n = 9)
	**100 dB(A)**	42.4 ± 2.4 (n = 6)	40.5 ± 1.9 (n = 6)	40.7 ± 1.9 (n = 5)	40.3 ± 2.4 (n = 7)
**2000 Hz**	**80 dB(A)**	40.3 ± 0.8 (n = 3)	39.5 ± 1.3 (n = 4)	41.1 ± 2.1 (n = 9)	40.9 ± 1.5 (n = 9)
	**85 dB(A)**	41.9 ± 4.0 (n = 2)	40.2 ± 3.1 (n = 4)	38.3 ± 1.4 (n = 2)	42.4 ± 2.4 (n = 6)
	**90 dB(A)**	40.6 ± 2.1 (n = 3)	40.6 ± 1.7 (n = 8)	41.0 ± 0.0 (n = 2)	41.2 ± 2.2 (n = 7)
	**95 dB(A)**	39.8 ± 0.0 (n = 2)	41.7 ± 2.3 (n = 16)	40.7 ± 2.3 (n = 5)	41.2 ± 2.2 (n = 12)
	**100 dB(A)**	43.1 ± 1.2 (n = 4)	40.9 ± 1.6 (n = 10)	40.4 ± 2.1 (n = 8)	41.1 ± 1.5 (n = 14)
**4000 Hz**	**80 dB(A)**	40.2 ± 1.4 (n = 4)	40.9 ± 2.2 (n = 14)	40.9 ± 1.0 (n = 18)	40.9 ± 1.1 (n = 24)
	**85 dB(A)**	40.3 ± 0.8 (n = 5)	41.4 ± 1.4 (n = 21)	41.5 ± 1.2 (n = 18)	40.7 ± 1.0 (n = 21)
	**90 dB(A)**	40.9 ± 1.1 (n = 6)	41.2 ± 1.2 (n = 16)	41.1 ± 1.2 (n = 20)	41.0 ± 1.2 (n = 29)
	**95 dB(A)**	41.7 ± 1.7 (n = 10)	41.3 ± 1.5 (n = 16)	41.2 ± 0.9 (n = 15)	41.2 ± 1.3 (n = 28)

SD = standard deviation, n = number

Supplementary Figures

Supplementary Figure S1. Hearing condition of the subjects. (A,B) show the results of the pure tone audiometry. (C,D) show the results of the stapedius reflex threshold.

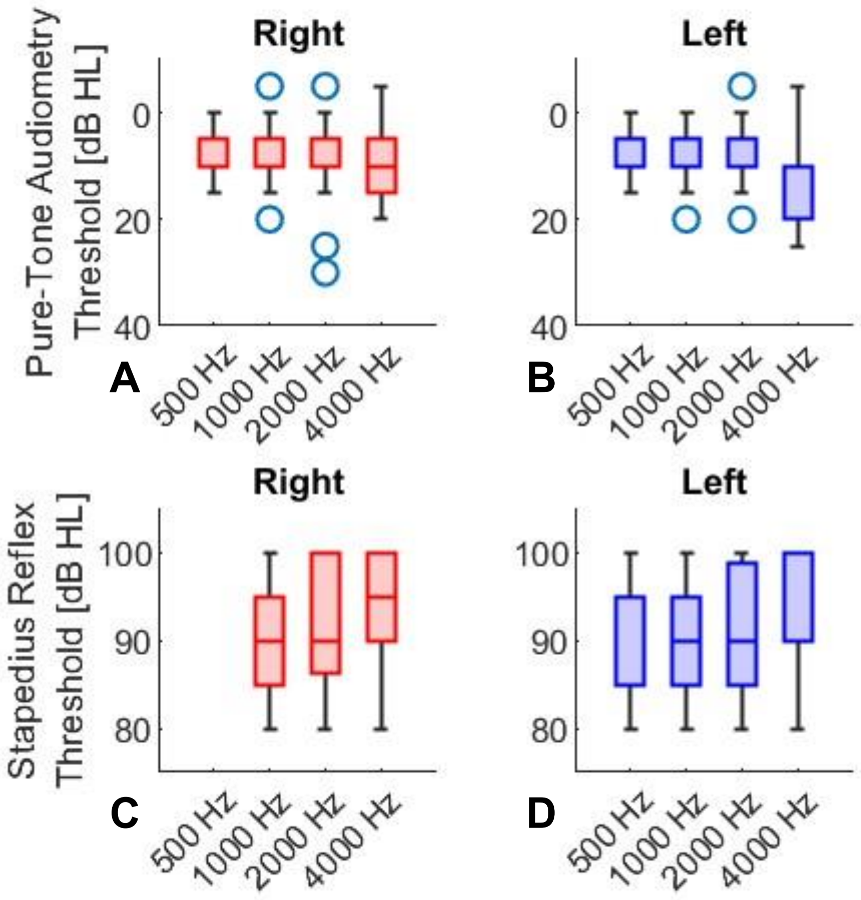

Supplementary Figure S2. Prevalence of the PAMR across the most significant variables.

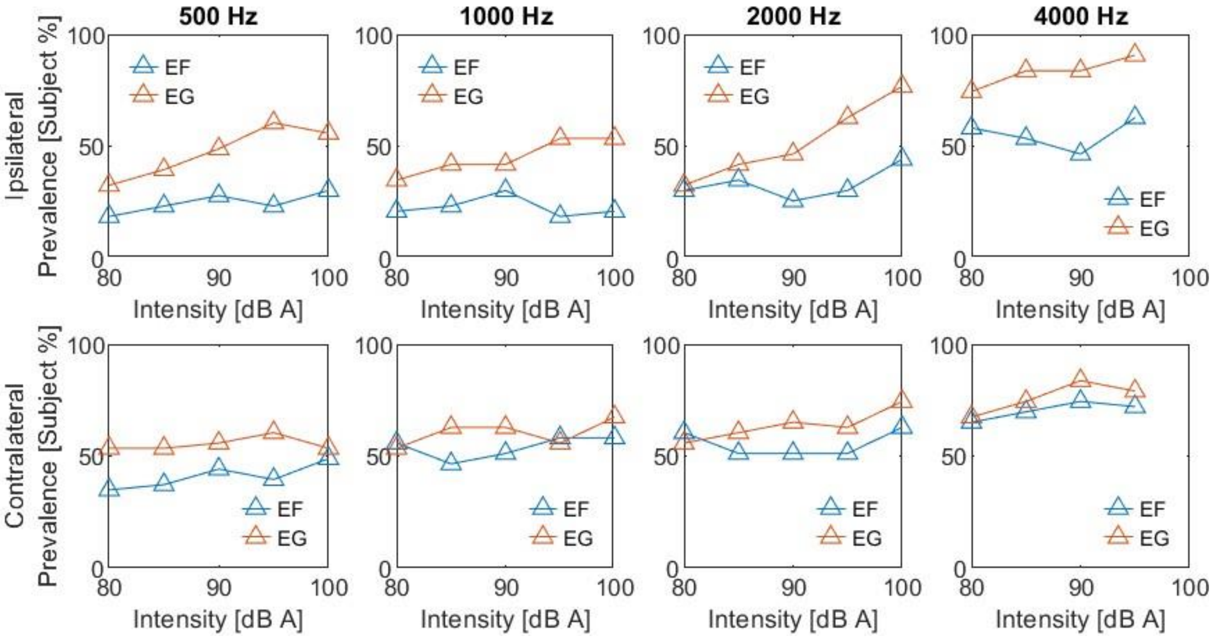

Supplement: Supplementary file 1 [file sensors-26-02524-s001.zip › sensors-4203700-supplementary.pdf]
